# Comparison of Gas Sensing Properties of Reduced Graphene Oxide Obtained by Two Different Methods

**DOI:** 10.3390/s20113175

**Published:** 2020-06-03

**Authors:** Sabina Drewniak, Marcin Procek, Roksana Muzyka, Tadeusz Pustelny

**Affiliations:** 1 Department of Optoelectronics, Silesian University of Technology, 2 Krzywoustego St., 44-100 Gliwice, Poland; Marcin.Procek@polsl.pl (M.P.); Tadeusz.Pustelny@polsl.pl (T.P.); 2 Department of Electronics Design, Mid Sweden University, 85170 Sundsvall, Sweden; 3 Institute for Chemical Processing of Coal, 1 Zamkowa St., 41-803 Zabrze, Poland; rmuzyka@ichpw.pl

**Keywords:** reduced graphene oxide, gas sensor, sensing structure, hydrogen

## Abstract

In this study, the sensitivity of reduced graphene oxide structures (rGO) to the action of selected gases (especially hydrogen, but also nitrogen dioxide and ammonia) was examined. Two sensing structures, based on rGO structures, obtained by different methods of oxidation (the modified Hummers, and the modified Tour’s method respectively), were investigated. We show here that the method used for the oxidation of rGO influences the sensitivity of the sensing structure during contact with various gaseous atmospheres. We performed our experiments in the atmosphere, containing hydrogen in a concentration range from 0 to 4% in nitrogen or synthetic air, both in dry and wet conditions. The temperature range was from 50 °C to 190 °C. Finally, we checked how the resistance of the samples changes when the other gases (NO_2_, NH_3_) appear in tested gas mixtures. The gas investigations were supplemented by the characterization of rGOs materials using scanning electron microscopy (SEM), X-ray diffraction (XRD), X-ray photoelectron spectroscopy (XPS) and N_2_ sorption method.

## 1. Introduction

The family of carbon materials, includes graphite (3D), graphene (2D), carbon nanotubes (1D) and fullerenes (0D) [1,2]. In recent years, the graphene attracted a great interest of researchers [3]. This material is characterized by a good mechanical properties [3,4], excellent electrical conductivity [3] and thermal properties [4,5]. In the literature, much attention is devoted to the properties of graphene, as well as the methods of its fabrication. Exemplary production methods are: Micromechanical cleavage, epitaxial growth or chemical vapour deposition methods [3]. Undoubtedly, this material is unique, but it is worth paying attention to. Besides the predominant number of carbon atoms, it also contains additional functional groups. An example could be the reduced graphene oxide (rGO). This material contains residual functional groups [5,6] and defects [5], while the carbon layers are exfoliating [6]. It is worth mentioning that the production of rGO is relatively easy and inexpensive [7]. The obtained product is also called chemically derived graphene, chemically modified graphene or reduced graphene [5].

rGO is prepared in several ways. Generally, this material is obtained by oxidation of graphite to graphite oxide (GO) and further reduction of such GO [8] (the advantage is that graphite is an inexpensive material [9]). The most popular oxidation methods, include Hoffmann [10], Staudenmaier [10,11], Tour [10,11] and Hummers [10,11]. These method differ among others in the use of chemical reagents (concentrated H_2_SO_4_ along with: HNO_3_ and KClO_3_ oxidant, fuming nitric acid and KClO_3_ oxidant, concentrated H_3_PO_4_ with mixture KNO_3_ + KMnO_4_ and NaNO_3_ and KMnO_4_ for Hoffmann, Staudenmaier, Tour, and Hummers method respectively [12,13]). The structural properties and degree of exfoliation depend on the degree of oxidation of graphite oxide (GO) [10], but at the same time, the reduction has also a significant impact on the properties of the final material (rGOs) and, of course, on the properties of the systems based on rGOs [5]. It is worth noting that many reduction methods are known, such as thermally [6] and photo-thermally process [6] or using the chemical reactions with strong reducing agents [8]. Reduced graphene oxide, with its high surface to volume ratio and a large number of defects, enables the adsorption and other catalytic activity to a greater extent than graphite oxide [14]. This allows composites with the electroactive materials to form, which could be used in electrochemical sensors [14]. In practice, however, both structures with bare reduced graphene oxides and hybridized with other materials are used in the gas sensing investigations [15,16,17,18,19]. An example could be the heterostructures presented in the paper [17]. The authors proposed the combination of (rGO and Au@SnO_2_) and (rGO and SnO_2_). They showed that such hybrid structure could detect an ammonia even in 25 °C. Moreover, it is characterized by fast response and recovery. An another example is proposed in [20]. The authors presented the structure with hybrid of reduced graphene oxide with gallium nitride nanorods (rGO/GaN NRs) for the detection of hydrogen. It is worth noting that such structure shows a higher response to hydrogen (and also to H_2_S) than bare gallium nitride. Yet another combination of materials is presented in [21]. J. Sun et al. show in their paper the results of investigation using 4.0 wt% rGO/CdS/CdO composite to detect a low concentration of NO_2_ in 125 °C. However, the detection of NO_2_ is possible using also unhybridized reduced graphene oxide. In the paper [16], the authors report the ability to detect NO_2_ using bare reduced graphene oxide at room temperature. They also check the reaction of such material with respect to the appearance of hydrogen in the surrounding atmosphere. Also, the authors of [22] have successfully used reduced graphene oxide to detect NO_2_. The other gas analytes could also be detected using rGOs. An example is acetone or isopropyl vapor [23].

However, there are a small amount of studies concerning the pure rGO structures interaction with such gases like hydrogen. To meet this, we want to present the results of our research focused on the use of pure rGOs in a hydrogen detection. In our previous research [24], we showed that the rGO structures are much more sensitive to hydrogen (which is highly flammable reducing gas [14]), on a low explosive limit concentration range (1–4%), than graphite oxides. It is worth investigating the influence of the rGO structures, obtained by using different methods (with different morphologies, residual functional groups concentration and pore sizes) to gases. This knowledge would be interesting from the point of view of hybrid materials, containing rGO, used as a gas receptors. A proper choice of rGO fabrication method may optimize the potential gas sensing properties.

In this paper, we present investigations on the gas sensing properties of rGO structures, obtained by two methods (modified Hummers’ and modified Tours), which characterization was presented in details in our previous paper [13] (the investigations has been developed for the purpose of this article). Both materials were investigated using scanning electron microscopy (SEM), N_2_ sorption, X-ray diffraction (XRD) and X-ray photoelectron spectroscopy (XPS) to show their chemical, structural and morphological differences. In order to show the differences of their gas sensing properties, an electrical response of structures with rGOs in various conditions were analysed. We also checked the gas selectivity of the above mentioned materials, we performed the experiments using two toxic gases [21,25]: nitrogen dioxide (NO_2_) and ammonia (NH_3_).

## 2. Materials and Methods

### 2.1. Materials

We used two types of reduced graphene oxides, marked as **Material 1** and **Material 2**, in the investigations. The samples differed only by oxidation method and were prepared as follows:

- **Material 1**—oxidation: Modification of Hummer’s method (Method 1), reduction: thermal reduction.
- **Material 2**—oxidation: Modification of Tour’s method (Method 2), reduction: thermal reduction.

Both materials were obtained from natural graphite (flake): MG 192 SINOGRAF, (Toruń, Poland) (1 g). In the first step, graphite powder was milled in a planetary ball mill and next sieved to particle sizes <20 μm. Next, it was oxidized using methods 1 or 2 (giving graphite oxide). In the last step, thermal reduction was performed (giving reduced graphene oxide).

To avoid misunderstandings, we’ve listed the markings we’ll talk about:

**H**—Hummers’ method;
**T**—modified Tour’s method;
**GO1**—graphite oxide prepared using Hummers’ method;
**GO2**—graphite oxide prepared using modified Tour’s method;
**TRGO1**—graphene oxide obtained after thermally reduction of GO1;
**TRGO2**—graphene oxide obtained after thermally reduction of GO2;
**Sample 1**—a measuring structure with TRGO1;
**Sample 2**—a measuring structure with TRGO2.

#### 2.1.1. Oxidation

The graphite oxides (**GO1** and **GO2**) were obtained using modification of so-called Hummers’ (H) and Tour’s (T) method. These methods differ in the used reagents, i.e., acids and oxidizing mixtures. In the H method, concentrated (95–97%) sulfuric acid (not mixed with the other acids) was used, while in the T method, a mixture of sulfuric and orthophosphoric acid was used. The mixture of sodium nitrate with potassium permanganate and potassium nitrate with potassium permanganate were used as a oxidizing mixtures in H, and T methods, respectively. The oxidation time was longer in the second method. Detailed weight ratios are given in the Table 1.

Depending on the chosen method, concentrated sulfuric H_2_SO_4_: 95–97% (PURE P.A. POCH, Gliwice, Poland) and orthophosphoric: 85% (PURE P.A., STANLAB, Lublin, Poland) acid was used. Mixtures of KMnO_4_ (PURE P.A., Sigma-Aldrich, Poznań, Poland) with NaNO_3_ (PURE P.A. POCH, Gliwice, Poland) and of KMnO_4_ with KNO_3_ (PURE P.A. POCH, Gliwice, Poland) were used as oxidants. The oxidation processes were carried out in a round bottom flask (equipped with a reflux condenser, thermometer and magnetic stirrer) placed in an ice bath. One gram of graphite and the appropriate oxidizing agent (depending on the procedure used) were placed in the flask. After that, KMnO_4_ was added in small portions (to keep the reaction temperature below 10 °C) with vigorous stirring. The reaction temperature was maintained in the range of 5–25 °C throughout the entire oxidation process. A yellow-green suspension was obtained after 2, and 5 h in Method H and T, respectively. After the reaction, 100 mL of deionized water and 60 mL of 3% H_2_O_2_: 30% (PURE P.A. POCH, Gliwice, Poland) solution were slowly added to the suspension, and then mixed for 30 min and centrifuged, using a laboratory centrifuge (5000 rpm, 0.5 h/cycle). The solution was then decanted, and the resulting precipitate was washed with 10% HCl: 35–38% (PURE P.A., CHEMPUR, Piekary Śląskie, Poland) solution. Next, the solution was centrifuged, and the resulting suspension was repeatedly washed with water (conductivity of 0.14–0.18 µS) until the pH was equal to 5. The graphite oxides were dried in a vacuum oven at 50 °C for 24 h.

#### 2.1.2. Exfoliation and Reduction

The reduced graphene oxides were obtained using thermally reduction. The obtained rGOs were marked as TRGO1 and TRGO2. The processes of such reduction (and exfoliation) were carried out as follows:

- The graphite oxide sample (10 g), after being placed in the retort, was purged with nitrogen (5 mL/min) at room temperature for 30 min;
- The retort with the sample was then placed in an oven preheated to 900 °C and kept at this temperature for 5 min.

Under these conditions, the gases resulting from the decomposition of oxygen functional groups were rapidly released, causing an increase in pressure, which in turn causes simultaneous exfoliation and reduction of graphene planes. The reduced graphene oxide was removed in a specifically designed receptacle.

### 2.2. Preparation of Sensing Structures

**Figure 1:**
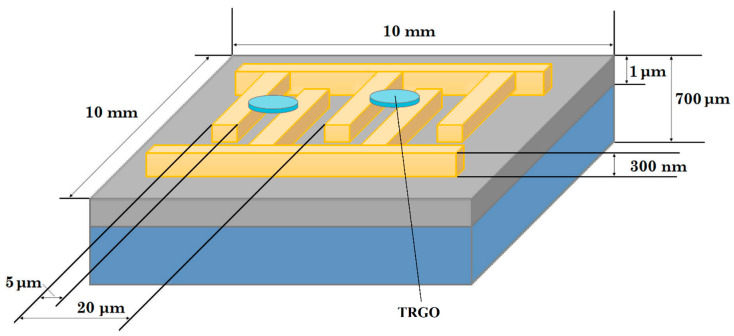
A simplified structure drawing.

The structures were prepared in the following way: (i) graphene oxides (reduced/exfoliated graphene oxide prepared as described above) were mixed with ethanol (ETHYL ALCOHOL ABSOLUT 99.8% PURE P.A., POCH, Gliwice, Poland) using ultrasonic stirring for 15 min; (ii) the suspensions were applied onto interdigital transducers (IDT), with gold electrodes (on 2 nm of chromium layer) on SiO_2_/Si substrate, schematically presented in the Figure 1, using the drop coating method; (iii) the structures were then annealed at 200 °C in dry N_2_ atmosphere.

The structures were electrically connected with chip feedthroughs using a 25 μm gold wire by the ultrasonic wire bonding method (wire bonder 53XX-BDA, F and K DELVOTEC, Braunau, Austria). The ready-made sensing structures are marked as **Sample 1** and **Sample 2**, depending on the receptor material used: TRGO1, and TRGO2, respectively.

### 2.3. Methods

#### 2.3.1. Gas Measurement System

**Figure 2:**
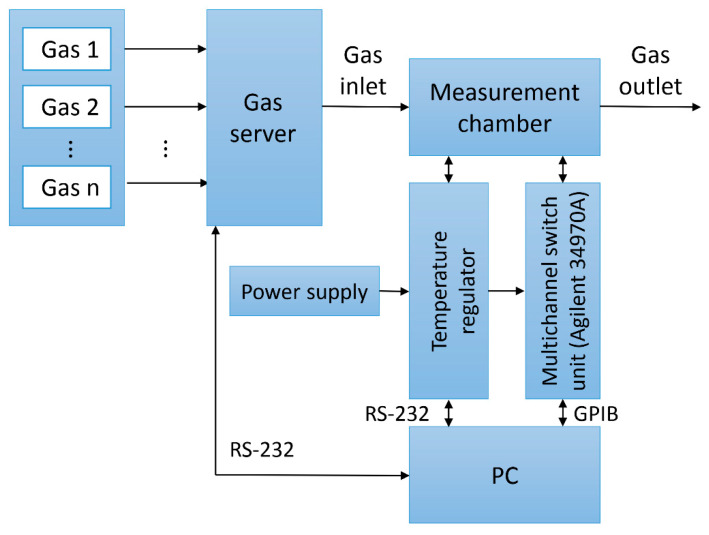
The scheme of the gas measuring system.

Resistance measurements were carried out at different gas mixtures composition, with different humidity level and different sample temperatures. The scheme of the gas measurement system is presented in Figure 2. The system enables to dosage a selected gas in a precise way and also creating the gas mixtures of the selected composition using gas server based on mass flow controllers. The gas was introduced into the measuring chamber with a stable gas flow of 500 mL/min. Several samples were placed in the chamber and the measurement were performed for each one at the same time (to preserve the same measurement conditions). The temperature of the samples was controlled by using a proportional-integral-derivative (PID) controller SR94 (Shimaden, Tokyo, Japan) while the resistance were measured using multi-switch unit 34970A (Agilent, Santa Clara, CA, USA). Data acquisition and measurement control was done using PC computer (LabVIEW software).

The mixtures with different concentrations of hydrogen, nitrogen dioxide and ammonia in a carrier gas (nitrogen or synthetic air) were used in the measurements. The gas humidity was maintained at one of two levels: 7 ± 1% (called as dry) and 50 ± 1% (called as wet) while the temperature was changed in the range from 50 °C to 190 °C.

#### 2.3.2. Porous Texture Analysis Using N_2_ Sorption

The textural parameters of reduced graphene oxides were determined by measuring N_2_ sorption at 77 K (−196 °C) using 3Flex^TM^ (company: Micromeritics, Norcross, GA, USA). Before the measurement, the samples were degassed at 350 °C for 24 h. Nitrogen adsorption and desorption isotherms were determined in the relative pressure range p/p_0_ = 0.01 to 0.96, which allowed us to determine the micro- and mesopore volume and the total pore volume. The surface area of the pores (S_BET_) was determined from Brunauer, Emmett and Teller (BET) equations. The total pore volume was calculated from the Gurvitch law based on the amount of adsorbed gas at a relative pressure p/p_0_ = 0.96. The average pore diameter (d_av_) was determined from the equation d_av_ = 4V_T_/S_BET_ [26].

#### 2.3.3. Scanning Electron Microscopy (SEM), X-ray Diffraction (XRD) and X-ray Photoelectron Spectroscopy (XPS)

The images of samples were obtained using a scanning electron microscopy (Inspect S50, FEI, Hillsboro, USA). The measurements using X-ray diffraction (X’Pert PRO PW 3040/60 diffractometer (PANalytical, Quebec, Canada)) were performed using CuKa1 radiation with a voltage of 45 kV and a current of 30 mA. The measurements using X-ray photoelectron spectroscopy (PHI 500 VersaProbe spectrometer from Chigasaki, Japan)) were performed using an Al Kα anode radiation beam (1486.6 eV).

## 3. Results

In this paper, we present the results of the measurements performed using the structures differing only in receptor layers (TRGO1/TRGO2). We characterized the morphology and chemical composition of both materials and performed the electrical measurements in various conditions (we placed both types of structures in one measuring chamber to maintain the same measuring conditions).

### 3.1. Characterization of the Structures

#### 3.1.1. Porous Texture Analysis Using the N_2_ Sorption Method

**Figure 3:**
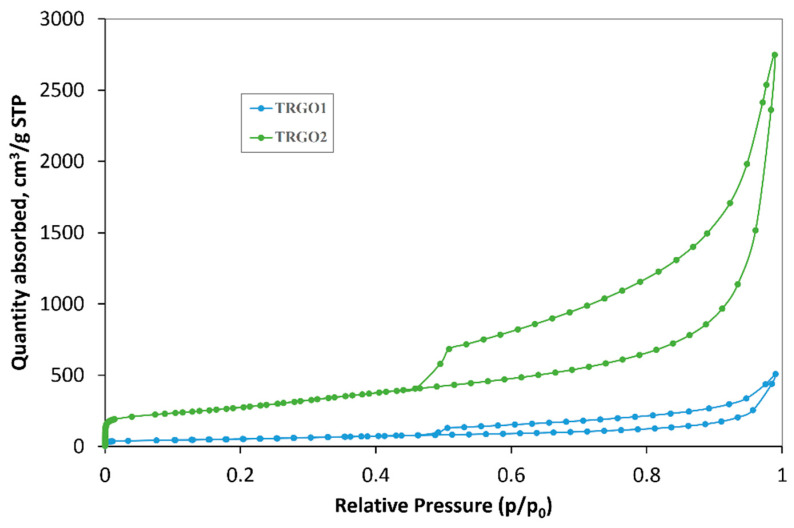
Isotherms of adsorption and desorption of N_2_ at 77 K (for Materials 1 and 2).

The porosity of TRGO1 and TRGO2 were investigated by the N_2_ sorption method at 77 K. Figure 3 shows the N_2_ sorption isotherms for these materials, and Table 2 gives the textural parameters determined on the basis of the isotherms.

The N_2_ sorption isotherms at 77 K for TRGO 1 and 2 are typical IV type isotherms with a characteristic hysteresis loop that indicates the presence of mesopores in the texture of the materials.

Exceptionally high surface area (S_BET_) values are obtained for TRGO2. For this material, the micropore volume and mesopore volume are approximately 5 times greater than for TRGO1. The high V_mez_/V_T_ ratios in a similar range confirm the dominant mesoporous nature of the reduced graphene oxides. The average pore diameter is the same for both materials. Based on the obtained data, the oxidation of flake graphite, using TRGO2, appears to be an attractive way of obtaining reduced graphene oxide with a large surface area and mesopore volume.

#### 3.1.2. Imaging of the rGO Structures

Both samples were characterized using SEM to determine how rGOs were arranged on the IDT transducers. In both cases, the embedded rGOs did not form a uniform layer, but agglomerates are formed and spread throughout the entire area. The diameter of the agglomerates was from 2 μm to 20 μm for both sensing materials.

**Figure 4:**
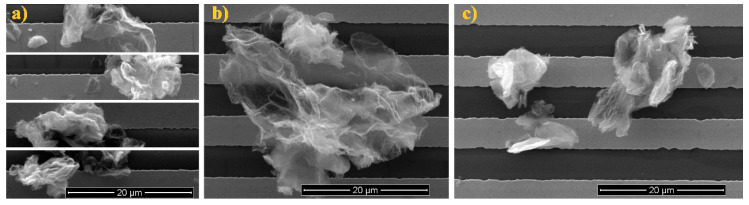
SEM images of graphene oxide: (**a**) Sample 1 (different areas), (**b**) Sample 2 (cloud type structure) and (**c**) Sample 2 (compact type structure).

Although the same structure application was used on IDT, the samples differed from each other. The agglomerates formed from TRGO1 have cloud-like structures (Figure 4a), which seems to be well packed. Part of the agglomerates formed from TRGO2 has also cloud type agglomerates (Figure 4b) but there are clearly looser than in the Sample 1. The second part of the agglomerates in Sample 2 are quite compact, weakly wrinkled and flakier than the others (Figure 4c).

#### 3.1.3. Composition Analysis Using X-ray Diffraction (XRD) and X-ray Photoelectron Spectroscopy (XPS)

The investigations using XPS and XRD were necessary to gather an information about the size of crystallites, number of layers in crystallites, interplanar distances and the number of carbon atoms with sp^2^ and sp^3^ hybridization, as well as the type (and the quantity) of the residual functional groups.

**Figure 5:**
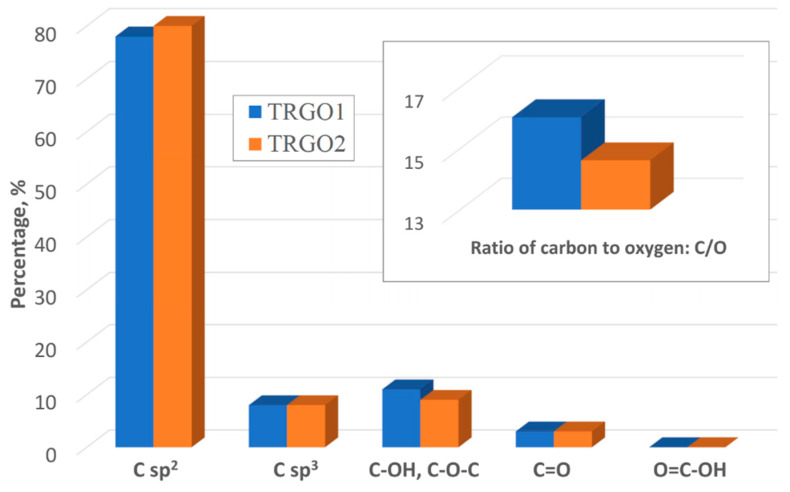
Distribution of carbon and oxygen bonds determined by XPS.

**Figure A1:**
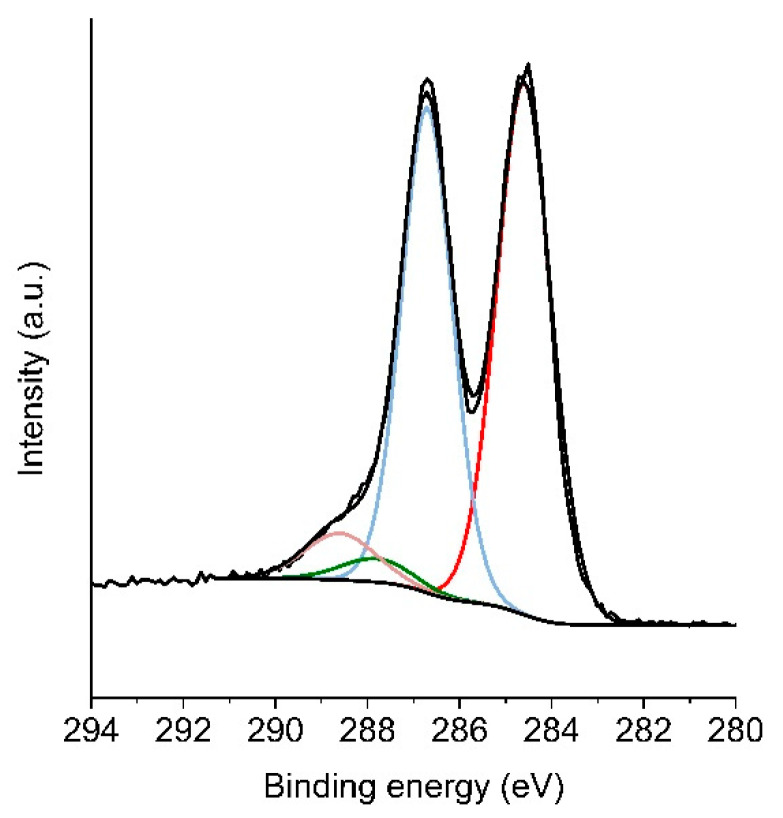
C1s spectra (XPS) of graphite oxide GO1.

**Figure A2:**
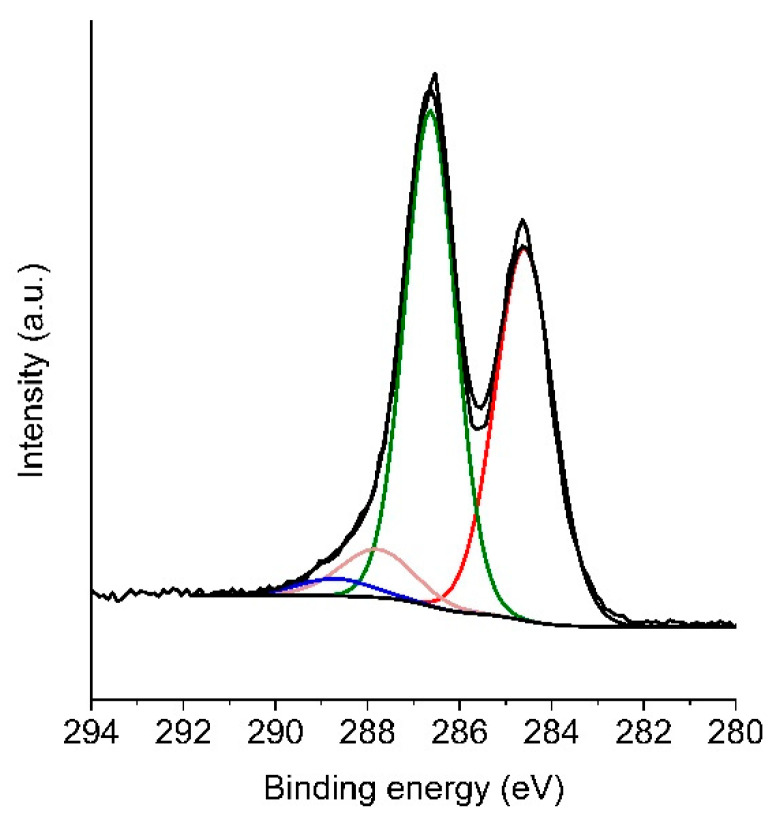
C1s spectra (XPS) of graphite oxide GO2.

**Figure A3:**
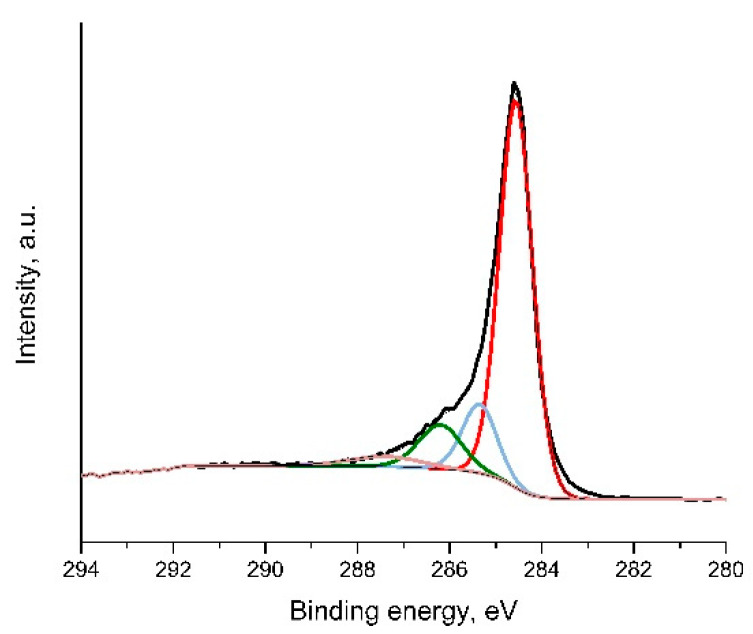
C1s spectra (XPS) of thermally reduced graphene oxide TRGO1.

**Figure A4:**
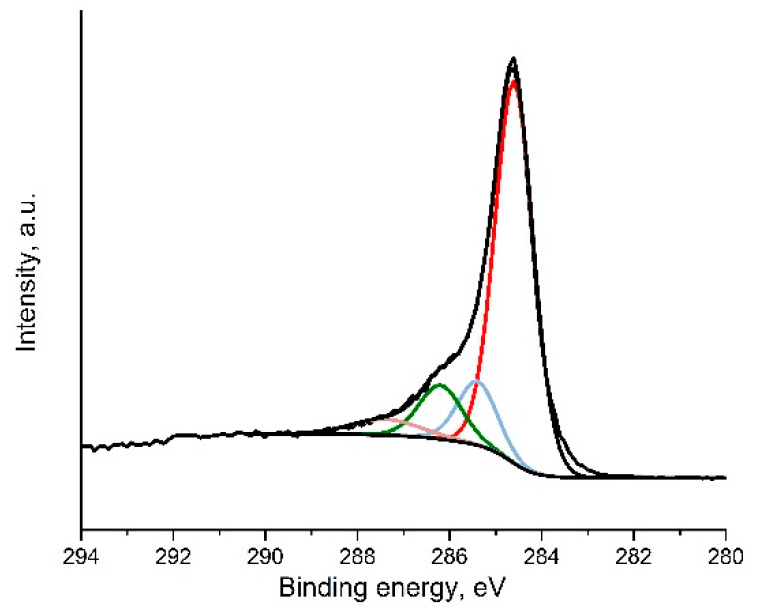
C1s spectra (XPS) of thermally reduced graphene oxide TRGO2.

The materials mostly consist of carbon atoms with sp^2^ hybridization (78% and 80% for TRGO1, and 2, respectively). The atoms with sp^3^ hybridization constitute 8% in each case. Moreover, both oxides consist C-OH and C-O-C in amount of 11% for TRGO1 and of 9% for TRGO2. The quantity of C=O groups is the same for both oxides (3%), while O=C-OH groups are not present in any oxide. The amount of carbon relative to oxygen is 16.02 and 14.61 for TRGO1 and 2 respectively. This data were graphically presented at Figure 5 (detailed data about distribution of carbon and oxygen bonds for graphite oxide/thermally reduced graphene oxide and narrow-scans of C1s are in the Appendix A, designated as Table A1 and Figure A1, Figure A2, Figure A3 and Figure A4, respectively).

**Figure 6:**
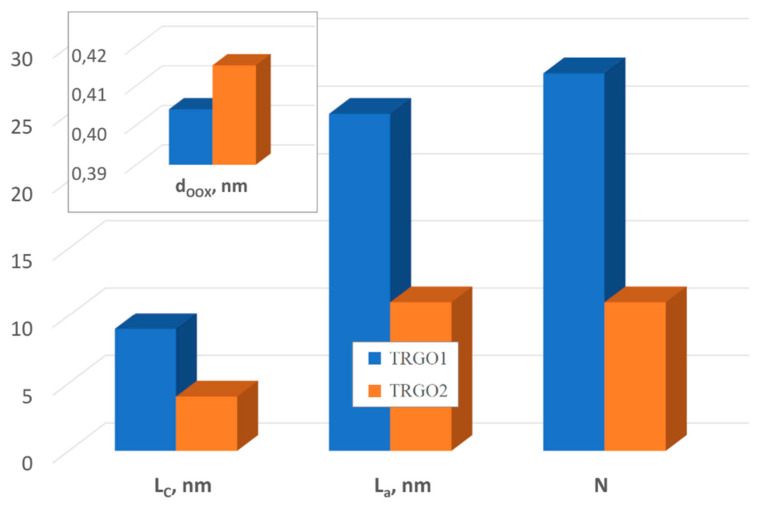
The structural parameters determined using XRD method.

**Figure A5:**
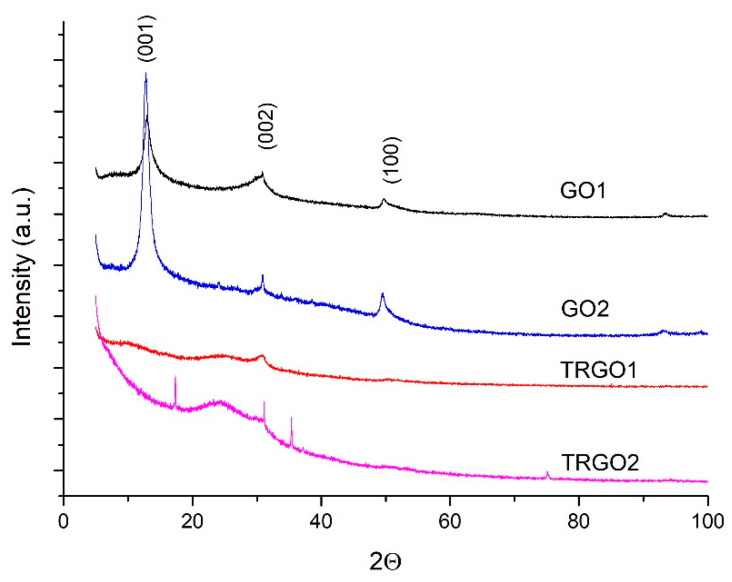
Diffractograms of graphite oxide and thermally reduced graphene oxide.

The crystallite diameter (L_a_), crystalline height (L_c_) and number of layers (N) in a crystallite (Figure 6) are smaller and the interplanar distance (d_00X_ band) are bigger in the case of TRGO2 (TRGO1: 25 nm, 9 nm, 28, 0.4040 nm, TRGO2: 11 nm, 4 nm, 11, 0.4151 nm). Diffraction patterns of graphite oxides and reduced oxides are in Appendix A: Figure A5.

The reduced graphene oxides, obtained using the Hummers’ method, are characterized by a larger diameter of crystallites that result in bigger surface areas (which adheres of such agglomerated to the substrate, i.e., the agglomerate holding force is greater and it is harder for such agglomerate to fall). This results in more agglomerates, obtained using modified the Hummers’ method, than those obtained by the modified Tour’s method after the application process, despite the same way of applying of both materials.

### 3.2. Study of the Sensitivity of Receptor Layers

#### 3.2.1. Hydrogen Action in Dry Carrier Gas on rGO Structures

The resistance of Samples 1 and 2 should depend both on the temperature and composition of the surrounding atmosphere. To confirm this, the pure carrier gas (nitrogen or synthetic air) and hydrogen at a concentration of 1% in a carrier gas was dosed. The measurements were performed at various temperatures. The data for calculations was taken as follows; first, a pure carrier gas (at first temperature; T_1_) was introduced to the measuring chamber (i), then the mixture of gases was introduced and we were waiting for the signal stabilization (ii), next we introduced a pure carrier gas again (iii). In the subsequent steps, the temperature was increased and the steps (i)–(iii) were repeated. It can be noticed that resistance is increasing with temperature. Similar behaviour is observed for the case of synthetic air and for both carrier gases for Sample 1.

To analyse the changes of resistance, caused by operating temperature, we have chosen the temperature range from 100 °C to 170 °C. The resistance of Sample 1 during dosing a pure nitrogen increased by 2.59 mΩ/°C, while the resistance of Sample 2 increased by 4.01 mΩ/°C. Analogous analyses were performed for the results obtained in pure synthetic air: The resistance of Sample 1 increased by 3.41 mΩ/°C, while the resistance of Sample 2 increased by 4.08 mΩ/°C. We also performed similar calculations for the data obtained in the experiments with mixtures of hydrogen (1%) in nitrogen and of hydrogen (1%) in synthetic air. The resistance of Sample 1 increased by 2.63 mΩ/°C, while the resistance of Sample 2 increased by 4.26 mΩ/°C during contact with a mixture of hydrogen/nitrogen. In hydrogen/synthetic air, the resistance increased by 3.48 mΩ/°C and 4.27 mΩ/°C for Samples 1 and 2, respectively.

The resistance of Sample 2 increased in a greater extent in all tested gases. For the TRGO used in this sample, the surface S_BET_, total pore volume and mesopore volume were several times larger than for TRGO1. It should also be noted that the residual oxygen groups were located on the surface of both samples. The amount of oxygen in relation to carbon was higher for TRGO2 (based on [13]). It can be assumed that the contact surface of the sample with the atmosphere has an impact on the obtained results.

It is also worth adding that the resistance measured during dosing the atmosphere containing hydrogen was slightly larger than during dosing pure carrier gas, regardless of the sample and the type of carrier gas.

In the next part of this paper, the sensitivity of the samples to the composition of the surrounding atmosphere is examined in more details. The registered resistances were calculated using Equation (1), giving the Response:

(1) $$\mathrm{Response}=\frac{\Delta R}{R}=\frac{R_{X}-R_{C}}{R_{C}}\times 100,\;\%$$

where:

R_X_- resistance of target gas (structure is affected by testing gas);
R_C_- resistance of the structure affected by carrier gas.

**Figure A6:**
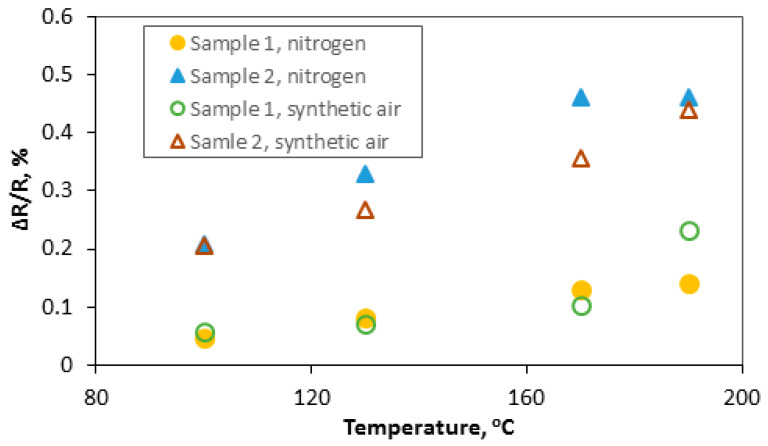
The response of Samples 1 and 2 affected by hydrogen in various temperatures.

On the basis of our earlier experiments, the authors concluded that the temperature range from 100 °C to 190 °C is the appropriate range for the analysed structures because the signal changes below 100 °C are small and the signal noise above 190 °C is significant. Both for nitrogen and synthetic air as a carrier gas, the ΔR/R increased with temperature (see also Appendix A: Figure A6). ΔR/R was several times larger for Sample 2, regardless of the type of carrier gas. The influence of the surface area and the possibility of transporting the carrier gas by mesopores is evident here: Sample 2 is characterized by a larger surface area and mesopore volume.

**Figure 7:**
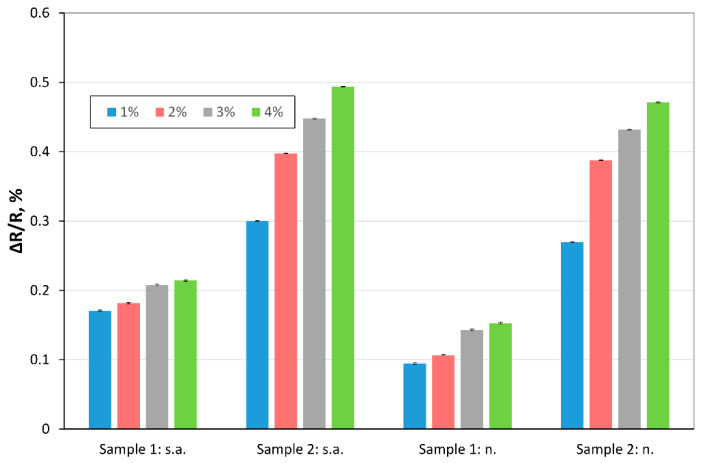
$\frac{\Delta R}{R}$ calculated for various concentration of hydrogen in synthetic air (“s.a.”) and nitrogen (“n.”), temperature: 150 °C.

**Figure 8:**
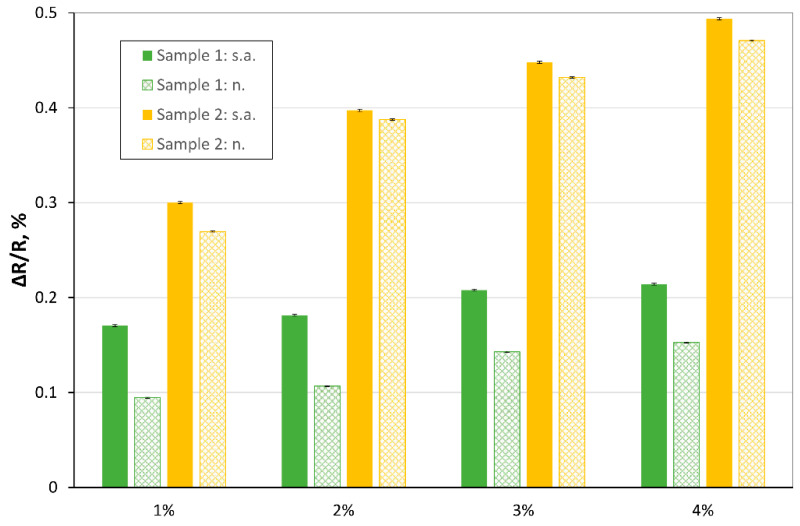
$\frac{\Delta R}{R}$ calculated for various concentration of hydrogen in synthetic air (“s.a.”) and nitrogen (“n.”), temperature: 150 °C.

In the next experiments, we investigated the response of the samples to different concentrations of hydrogen (in the range of: 0–4%) in carrier gas. The measurements were performed at various temperatures. The ΔR/R vs. concentration of hydrogen is presented Figure 7 and Figure 8 (for 150 °C).

The use of a mixture of acids in Tour’s method, including orthophosphoric acid, resulted in a higher surface area of TRGO2 than TRGO1 (obtained by Hummers’ method using only sulfuric acid). The presence of functional groups in both materials is similar (slightly larger for Sample 1), but the surface of interaction with the gas is much bigger in TRGO2, which results in greater sensitivity (obtaining a higher value of the ΔR/R for Sample 2) as a result of contact with hydrogen. Figure 8 clearly presents that in the case of using nitrogen as a carrier gas, the ΔR/R_Sample 2_ is 2.5 to 3 times bigger than ΔR/R_Sample 1_ while in the case of synthetic, the ΔR/R_Sample 2_ is ~2 times bigger than ΔR/R_Sample 1_, for 150 °C. As shown in the above figures, ΔR/R increases with increasing hydrogen concentration. The hydrogen concentration can be estimated, based on its value.

#### 3.2.2. Hydrogen Action in Wet Carrier Gas on rGO Structures

**Figure 9:**
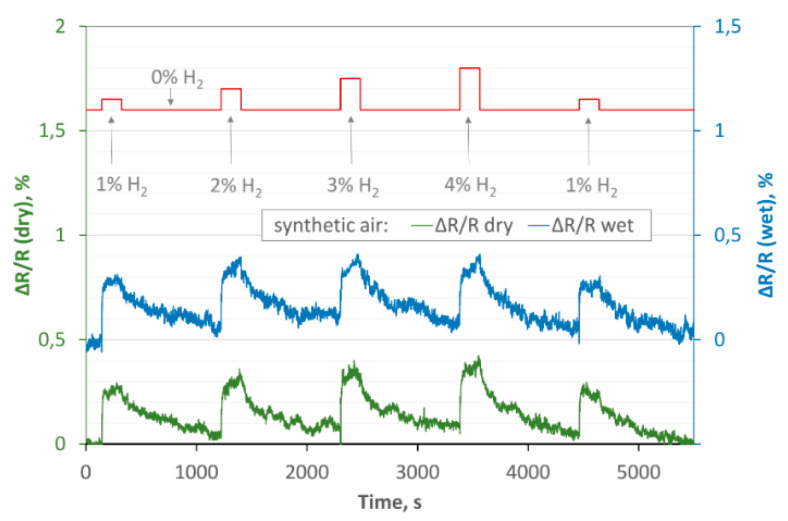
ΔR/R calculated for dry and wet synthetic air vs. time, temperature: 150 °C, concentration of hydrogen in synthetic air, Sample 2.

In further investigations, we analysed the sensitivity of the structures in dry (~7% humidity) and wet (~50%) carrier gas. As the experiments showed, Samples 1 and 2 are poorly sensitive to changes in the humidity of the surrounding atmosphere. It can be seen in the Figure 9 (which shows the results obtained for Sample 2 at 150 °C, carrier gas: Synthetic air) in the form of a real course. Even though the humidity varies significantly, the obtained results for dry and wet gas do not differ much from the each other.

To check to what extend the humidity affect the obtained results, we calculated the P_ch_ using Equation (2). P_ch_ is informing about the ratio of ∆R/R_dry_ to ∆R/R_wet_. The obtained results for Sample 2 at 150 °C (carrier gas: synthetic air) are presented in Table 3.

(2) $$P_{\mathrm{ch}}=\frac{{\left(\frac{\Delta R}{R}\right)}_{\mathrm{wet}}}{{\left(\frac{\Delta R}{R}\right)}_{\mathrm{dry}}}$$

In addition, by analysing the data presented in Figure 9, it can be seen that the results are reproducible. We dosed hydrogen in a dry carrier gas in the following sequence of concentrations: 0, 1, 0, 2, 0, 3, 0, 4, 0, 1, 0% (we checked the stability of the results by repeating the measurements with 1% hydrogen at the end of the cycle). The obtained value for 1% hydrogen measured at the beginning and at the end of the dosing cycle is comparable.

In the following analysis, we also investigated the reaction time t_rc_ (from pure carrier gas to gas mixture) and regeneration time t_reg_ (from gas mixture to pure carrier gas), understood as the difference between the times in which the resistance reaches 10 and 90% of its stabilized value. The exemplary values are shown in the Table 4.

Based on the registered data, it can be concluded that:

- for the same concentration of hydrogen and the same temperature but different humidity of the carrier gas, the t_rc_ times are comparable (for each sample);
- the t_rc_ is slightly shorter for Sample 2;
- t_reg_ is much longer than t_rc_, for all cases.

#### 3.2.3. Ammonia and Nitrogen Dioxide Action on rGO Structures

**Figure 10:**
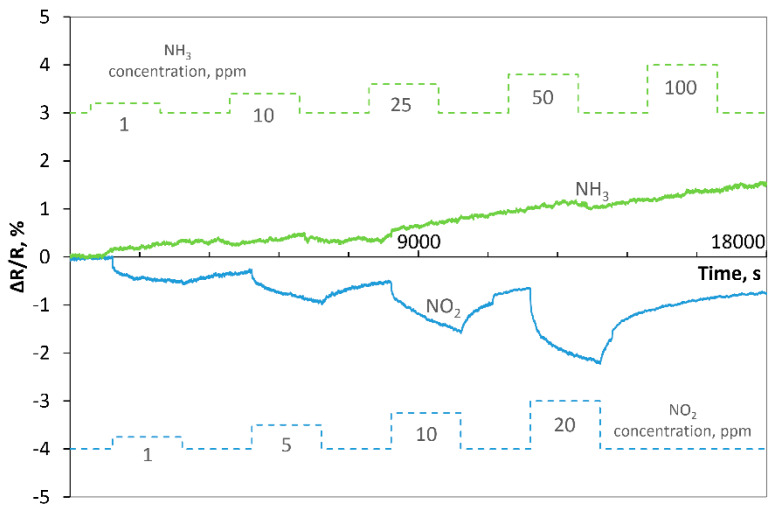
Response of Sample 2 to NH_3_ and NO_2_ at 150 °C.

**Figure A7:**
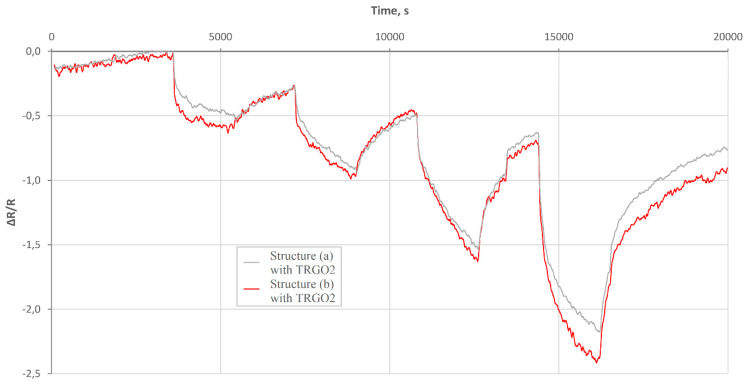
An exemplary response of two samples (with the same thermally reduced graphene: TRGO2) vs. time, temperature: 150 °C.

We performed additional experiments to review the sensitivity of the samples for two other gases, ammonia (NH_3_) and nitrogen dioxide (NO_2_), at 150 °C. The experiments were performed for various concentrations (5, 10, 25, 50 and 100 ppm) of ammonia in dry synthetic air. Both samples were slightly sensitive to ammonia in the ppm range; there were no evident changes in the resistance, but rather its insignificant increase similarly to the baseline drift. Completely reverse reactions of the samples compared to reducing gases (H_2_- Figure 9 and NH_2_- Figure 10) were registered during the reaction to oxidizing gas (NO_2_ at concentrations of: 1, 5, 10 and 20 ppm). When nitrogen dioxide was introduced into the atmosphere, the resistance of samples decreased, but the changes were much slower than in the case of H_2_. For example, for 1 ppm NO_2_ in dry synthetic air, the time t_rc_ estimated for Sample 1 was approximately equal 960 s, while for Sample 2, it was 1058 s. The regeneration time t_reg_ was longer for both structures (>30 min). For all tested concentrations, changes in the response (ΔR/R) were higher for Sample 2. The comparison of the results obtained for two structures with the same thermally reduced graphene oxide (TRGO2) is given in the Appendix A as Figure A7. This statement is intended to show the reproducibility of the method.

## 4. Discussion

As shown above, the interaction between reduced graphene oxides and hydrogen occurs, but the change of resistance is relatively low (response does not exceed 0.5%). This can be explained by two likely reasons. Firstly, hydrogen occurs in the form of a diatomic molecule (H2) and is the most stable molecule under normal and chemically unreactive conditions. Due to its small size, hydrogen easily penetrates through porous materials. Based on our research, it can be seen that the response of Sample 2 (i.e., structure with TRGO2 which is about five times more porous compared to TRGO1, and which has a bigger interplanar distance (the d00X parameter informs about it)) was greater, regardless of the type of carrier gas. Secondly, it seems that thermal reaction caused by gas-sample interaction is also significant. The thermal conductivity of hydrogen is several times higher than nitrogen or synthetic air, and hydrogen takes heat from the structures more efficiently. Even if the transducer is kept at a constant temperature, the thermally reduced graphene oxide (which is on it) is cooled down, and the sample resistance increases. It is worth mentioning that there is not much difference whether hydrogen is in the atmosphere of synthetic air or nitrogen - both gases have very similar thermal conductivity, which can be seen in the measurements.

Nitrogen dioxide had a much greater impact on the structure response than hydrogen. It should be noted that NO_2_ is a strong oxidizer [27]. Electrons are transferred from reduced graphene oxide to adsorbed NO_2_, and the whole concentration of rGOs is increased (the local carrier concentration is changed). Because of this, there is an improvement in the electrical conduction of rGOs [4,27].

As mentioned above, we have observed a very weak structure reaction to ammonia in the ppm concentration range. This results from a very poor reaction of reduced graphene oxide to NH_3_ and a lack of the recovery in pure carrier gas (it is shown in literature [27] that even for 1% of NH_3_, reaction of rGO is relatively low and its regeneration is extremely slow). It is widely known that the ammonia has sp^3^ hybridization, has a free electron pair and it is a highly polar molecule. It is also known that thermal reduction has removed most of these groups (what is confirm in the Table A1). As shown in [28], NH_3_ reacts with graphene oxide via oxygen groups (carboxyl, hydroxyl, epoxy groups). The reduction of number of functional groups decreases the polarity of obtained material, and reduces the number of potential active sites for NH_3_ adsorption what caused weak responses of TRGO structures. As this interaction involves a chemical adsorption of the NH_3_ on residual oxygen groups, its bonding to TRGO structures is strong and its recovery practically does not occur in examined conditions.

Our results shows that both tested TRGO structures have limited sensitivity to the tested gases (even though that Sample 2 is characterized by a bigger ΔR/R calculated for the experiments with hydrogen and nitrogen dioxide than Sample 1). This shows that thermally reduced graphene oxides are rather weak gas sensing materials, but such oxides may be functionalized or could be an element of more complex sensing structures.

Th analyses presented in the literature show that defects in graphene structure can improve gas adsorption on graphene. To increase this sensitivity, a good solution would be to artificially introduce defects and admixtures into its structure [29] (our results shows that proper choice of synthesis/reduction method has an effect on the chemical structure). Broadly understood doping of reduced graphene oxides, with other materials, can be realized both, at the stage of obtaining the oxides and by combining ready rGO with other materials. A good idea seems to be performing the reduced graphene oxide doped with palladium [30] or combined with more materials (e.g., polyaniline (PANI) and palladium [31]. In this case, palladium will play a key role. Contact with hydrogen will produce palladium hydride [30,31], and the adsorbed molecules will act as electron scattering centers, resulting in reduced carrier mobility (increased resistance) [31]. The investigations clearly prove that doped reduced graphene oxide will allow quite good responses to be obtained when the structure is in contact with hydrogen, even at room temperature (but also at higher ones, e.g., the response at 125 °C is about 38%) [31].

A completely different (and very promising) direction of modification/joining seems to be the combination of reduced graphene oxides with metal oxides (e.g., ZnO). The introduction of hydrogen in the surrounding atmosphere will generate a metallic Zn layer, which will result in the appearance of potential barriers in rGO/Zn as well as Zn/ZnO. The first potential barrier (rGO/Zn) prevents the electron flow into rGO. Therefore, the electrons from ZnO will not move to the rGO. This is the reason behind the decreasing resistance [32]. It is not an isolated opinion. The authors in [29] indicate that the systems, metal oxide/graphene can significantly improve electrical conductivity and sensor’s properties.

## 5. Conclusions

We investigated the sensitivity ofstructures with two differently reduced graphene oxides to various compositions of the surrounding atmosphere. We focused mainly on experiments with various concentrations of hydrogen in a carrier gas (synthetic air/nitrogen) in two different humidity level. Next, we supplemented our investigations by measurements with two other gases: ammonia and nitrogen dioxide. 

Based on the obtained results, we found that the undoubted advantage of both samples include:

- fast changes in resistance as a result of the presence of hydrogen in nitrogen/synthetic air (much shorter than for nitrogen dioxide);
- lack of sensitivity or poor sensitivity to humidity (an unquestionable advantage of both structures);
- selectivity: different reactions of the samples when mixture with hydrogen, ammonia or nitrogen dioxide are introduced to the atmosphere ($\frac{\Delta R}{R}$ is positive for the measurement with hydrogen, negative for the measurement with nitrogen dioxide and there is no reaction when NH_3_ was dosed). Our results are consistent with the literature which says that the structure can interact with NO_2_, but the interaction with NH_3_ is weak [29]. The absolute changes of the responses are much larger when in contact with NO_2_ than H_2_, for both structures.
- due to the potential sensor’s application of structures, the research was carried out at relatively low temperatures (i.e., 150 °C).

In summary, our research has shown that structures with reduced graphene oxides change their resistance as a result of contact with hydrogen and nitrogen dioxide. At the same time, the structures were characterized by a certain selectivity, showing different reactions (increasing or decreasing the resistance, different reaction time) to the presence of various gases in the atmosphere. According to our analyses, the structures with thermally reduced graphene oxide, obtained using the modified Tour’s method, were characterized by greater changes in $\frac{\Delta R}{R}$ ratios, than structures with thermally reduced graphene oxide obtained using modified Hummers’ method. In our view, structures with bare (not hybridized, undoped) reduced graphene oxides should be considered only as testing. There are necessary for the further experiments using a modified materials.

## Figures and Tables

**Table 1:** Chemical reagents used in the oxidation process.

Method of Oxidation	Reagents	Time, h	Obtained Graphite Oxide
H	H_2_SO_4_ (30 mL); NaNO_3_ (3 g); KMnO_4_ (3 g)	2	GO1
T	H_2_SO_4_ (45 mL); H_3_PO_4_ (5 mL); KNO_3_ (1.5 g); KMnO_4_ (5 g)	5	GO2

**Table 2:** The parameters of the porous structure of graphene oxides obtained using Methods 1 and 2.

Material	Surface Area (S_BET_) m^2^ g^−1^	Total Pore Volume (V_T_) cm^3^ g^−1^	Micropore Volume (V_DR_) cm^3^ g^−1^	Mesopore Volume (V_mez_) cm^3^g^−1^	V_mez/_V_T_	Average Pore Diameter (d_av_) nm
TRGO1	183 ± 13	0.41	0.06	0.35	0.87	9.6
TRGO2	965 ± 39	2.32	0.29	2.05	0.88	9.6

**Table 3:** The percentage change P_ch_ calculated for 150 °C, Sample 2, carrier gas: synthetic air.

Concentration of Hydrogen, %	Pch
1	0.9402 ± 0.0025
2	0.9213 ± 0.0020
3	0.9667 ± 0.0019
4	1.0297 ± 0.0020

**Table 4:** The reaction and regeneration times (mixture of 0–1% of hydrogen in synthetic air), temperature 150 °C (± 0.57 s).

	t_rc_ Dry, s	t_rc_ Wet, s	t_reg_ Dry, s	t_reg_ Wet, s
Sample 1	22	21	527	481
Sample 2	17	16	594	564

## Appendix A

**Table A1:** Distribution of carbon and oxygen bonds both for graphite oxide and thermally reduced graphene oxide by XPS, %.

Material	Csp^2^ 284.5 ± 0.1 eV	Csp^3^ 285.4 ± 0.2 eV	C-OH, C-O-C 286.5 ± 0.3 eV	C=O 287.6 ± 0.2 eV	O=C-OH 288.9 ± 0.3 eV
**GO1**	46	3	39	7	5
**GO2**	41	1	46	10	2
**TRGO1**	78	8	11	3	0
**TRGO2**	80	8	9	3	0

## References

[1] Ferrari A.C. (2007). Raman spectroscopy of graphene and graphite: Disorder, electron-phonon coupling, doping and nonadiabatic effects. Solid State Commun..

[2] Yang G., Li L., Lee W.B., Ng M.C. (2018). Structure of graphene and its disorders: A review. Sci. Technol. Adv. Mater..

[3] Peng X., Wu Y., Chen N., Zhu Z., Liu J., Wang H. (2019). Facile and highly efficient preparation of semi-transparent, patterned and large-sized reduced graphene oxide films by electrochemical reduction on indium tin oxide glass surface. Thin Solid Films.

[4] Geeta Rani B., Sai Bhargava Reddy M., Kailasa S., Maseed H., Bikshalu K., Venkateswara Rao K. (2019). Comparative gas sensing analysis of green and chemically reduced graphene oxide. Mater. Res. Express.

[5] Pei S., Cheng H.M. (2012). The reduction of graphene oxide. Carbon N. Y..

[6] Yar A., Dennis J.O., Mohamed Saheed M.S., Mohamed N.M., Irshad M.I., Mumtaz A., Jose R. (2020). Physical reduction of graphene oxide for supercapacitive charge storage. J. Alloys Compd..

[7] Sa K., Mahanandia P. (2019). Conducting reduced graphene oxide film as transparent electrode. Thin Solid Films.

[8] De Lima B.S., Bernardi M.I.B., Mastelaro V.R. (2020). Wavelength effect of ns-pulsed radiation on the reduction of graphene oxide. Appl. Surf. Sci..

[9] Wei M., Qiao L., Zhang H., Karakalos S., Ma K., Fu Z., Swihart M.T., Wu G. (2017). Engineering reduced graphene oxides with enhanced electrochemical properties through multiple-step reductions. Electrochim. Acta.

[10] Jankovský O., Marvan P., Nováček M., Luxa J., Mazánek V., Klímová K., Sedmidubský D., Sofer Z. (2016). Synthesis procedure and type of graphite oxide strongly influence resulting graphene properties. Appl. Mater. Today.

[11] Sali S., Mackey H.R., Abdala A.A. (2019). Effect of graphene oxide synthesis method on properties and performance of polysulfone-graphene oxide mixed matrix membranes. Nanomaterials.

[12] Chng E.L.K., Pumera M. (2013). The Toxicity of Graphene Oxides: Dependence on the Oxidative Methods Used. Chem. Eur. J..

[13] Muzyka R., Drewniak S., Pustelny T., Chrubasik M., Gryglewicz G. (2018). Characterization of graphite oxide and reduced graphene oxide obtained from different graphite precursors and oxidized by different methods using Raman spectroscopy. Materials.

[14] Arora K., Srivastava S., Solanki P.R., Puri N.K. (2019). Electrochemical Hydrogen Gas Sensing Employing Palladium Oxide/Reduced Graphene Oxide (PdO-rGO) Nanocomposites. IEEE Sens. J..

[15] Park J., Kim Y., Park S.Y., Sung S.J., Jang H.W., Park C.R. (2020). Band gap engineering of graphene oxide for ultrasensitive NO_2_ gas sensing. Carbon N. Y..

[16] Guo L., Hao Y.W., Li P.L., Song J.F., Yang R.Z., Fu X.Y., Xie S.Y., Zhao J., Zhang Y.L. (2018). Improved NO_2_ Gas Sensing Properties of Graphene Oxide Reduced by Two-beam-laser Interference. Sci. Rep..

[17] Peng R., Li Y., Liu T., Sun Q., Si P., Zhang L., Ci L. (2019). Reduced graphene oxide/SnO_2_@Au heterostructure for enhanced ammonia gas sensing. Chem. Phys. Lett..

[18] Al-Graiti W., Foroughi J., Liu Y., Chen J. (2019). Hybrid Graphene/Conducting Polymer Strip Sensors for Sensitive and Selective Electrochemical Detection of Serotonin. ACS Omega.

[19] Lee K., Yoo Y.K., Chae M.S., Hwang K.S., Lee J., Kim H., Hur D., Lee J.H. (2019). Highly selective reduced graphene oxide (rGO) sensor based on a peptide aptamer receptor for detecting explosives. Sci. Rep..

[20] Reddeppa M., Park B.-G., Kim M.-D., Rao K., Duc N., Kim D., Kim S.-G., Murali G. (2018). H_2_, H_2_S gas sensing properties of rGO/GaN nanorods at room temperature: Effect of UV illumination. Sens. Actuators B Chem..

[21] Sun J., Chen A., Sun L., Han N., Chu H., Bai S., Shu X., Luo R. (2019). rGO decorated CdS/CdO composite for detection of low concentration NO_2_. Sens. Actuators B Chem..

[22] Sharma N., Sharma V., Vyas R., Kumari M., Kaushal A., Gupta R., Sharma S.K., Sachdev K. (2019). A new sustainable green protocol for production of reduced graphene oxide and its gas sensing properties. J. Sci. Adv. Mater. Devices.

[23] Milovanov Y.S., Skryshevsky V.A., Slobodian O.M., Pustovyi D.O., Tang X., Raskin J.P., Nazarov A.N. Influence of Gas Adsorption on the Impedance of Graphene Oxide. Proceedings of the 2019 IEEE 39th International Conference on Electronics and Nanotechnology, ELNANO 2019- Proceedings (ELNANO).

[24] Drewniak S., Muzyka R., Stolarczyk A., Pustelny T., Kotyczka-Morańska M., Setkiewicz M. (2016). Studies of reduced graphene oxide and graphite oxide in the aspect of their possible application in gas sensors. Sensors.

[25] Biring S., Sadhu A.S., Deb M. (2019). An Effective Optical Dual Gas Sensor for Simultaneous Detection of Oxygen and Ammonia. Sensors.

[26] Barrett E.P., Joyner L.G., Halenda P.P. (1951). The Determination of Pore Volume and Area Distributions in Porous Substances. I. Computations from Nitrogen Isotherms. J. Am. Chem. Soc..

[27] Lu G., Ocola L.E., Chen J. (2009). Reduced graphene oxide for room-temperature gas sensors. Nanotechnology.

[28] Zhu S., Sun H., Liu X., Zhuang J., Zhao L. (2017). Room-Temperature NH_3_ sensing of graphene oxide film and its enhanced response on the laser-Textured silicon. Sci. Rep..

[29] Tian W., Liu X., Yu W. (2018). Research Progress of Gas Sensor Based on Graphene and Its Derivatives: A Review. Appl. Sci..

[30] Yatskiv R., Grym J. (2013). Hydrogen sensing using reduced graphene oxide sheets supported by Pd nanoparticles. J. Phys. Conf. Ser..

[31] Zou Y., Wang Q., Xiang C., Tang C. (2016). Doping composite of polyaniline and reduced graphene oxide with palladium nanoparticles for. Int. J. Hydrog. Energy.

[32] Abideen Z.U., Kim H.W., Kim S.S. (2015). An ultra-sensitive hydrogen gas sensor using reduced graphene oxide-loaded ZnO nanofibers. Chem. Commun..

